# Reviewer acknowledgement 2012

**DOI:** 10.1186/1465-9921-14-12

**Published:** 2013-02-28

**Authors:** Jan Lötvall, Reynold A Panettieri

**Affiliations:** 1 Göteborg University, Vasagatan 33, 411 37, Göteborg, Sweden; 2 University of Pennsylvania, 423 Guardian Dr #175, Philadelphia, PA, 19104, USA

## Abstract

**Contributing reviewers:**

The editors of *Respiratory Research* would like to thank all of our reviewers who have contributed to the journal in Volume 13 (2012).

## 

Jalal Abu-Shaweesh

United States of America

John Adcock

United Kingdom

Kenneth Adler

United States of America

Ioana Agache

Romania

Alvar Agusti

Spain

Dorothy Ainsworth

United States of America

John Alcorn

United States of America

Neil Alexis

United States of America

Sharilyn Almodovar

United States of America

Steven An

United States of America

Vikas Anathy

United States of America

Wayne Anderson

United States of America

Masaru Ando

Japan

Ignacio Ansotegui

Spain

Sabina Antoniu

Romania

Keisuke Aoe

Japan

David Aronoff

United States of America

Kewal Asosingh

United States of America

Baroukh Mautice Assael

Italy

Eric Austin

United States of America

Malin Axelsson

Sweden

Hoeke Baarsma

Netherlands

Gregory Bagby

United States of America

Chunxue Bai

China

Katherine Baines

Australia

Els Bakker

Netherlands

John Balmes

United States of America

Peter Barlow

United Kingdom

Eric Bateman

South Africa

Salvatore Battaglia

Italy

Richard Beasley

New Zealand

Michael Beers

United States of America

Juergen Behr

Germany

Abdelmadjid Belkadi

United States of America

Maria Belvisi

United Kingdom

Yves Berthiaume

Canada

Ivan Bertoncello

Australia

Urvashi Bhan

United States of America

Colin David Bingle

United Kingdom

Anders Bjerg

Sweden

Leif Bjermer

Sweden

Thomas Blankenburg

Germany

Yury Bochkov

United States of America

Vijay Boggaram

United States of America

Marcel Bonay

France

Diana Bonderman

Austria

Francesco Bonella

Germany

James Bonner

United States of America

Philippe Bonniaud

France

Michael Borchers

United States of America

Sixten Borg

Sweden

Apostolos Bossios

Sweden

Jean Bousquet

France

Ken Bracke

Belgium

Fulvio Braido

Italy

Caroline Bret

France

Steven Brody

United States of America

Cynthia Brown

United States of America

E Sherwood Brown

United States of America

Vito Brusasco

Italy

Guy Brusselle

Belgium

Pierre-Regis Burgel

France

Sergio Burguete

United States of America

Peter Calverley

United Kingdom

Carlos Camargo

United States of America

Gianna Camiciottoli

Italy

Matthew Campen

United States of America

Luigi Camporota

United Kingdom

Vera L Capelozzi

Brazil

Gaetano Caramori

Italy

Troy Carlo

United States of America

Chris Carlsten

Canada

Ciro Casanova

Spain

Didier Cataldo

Belgium

Pascal Chanez

France

Peter Chen

United States of America

Yin Chen

United States of America

Lee-Wei Chen

Taiwan

Chung-Ming Chen

Taiwan

Jianlin Cheng

United States of America

Donavan Cheng

United States of America

Myung-Haing CHO

Korea, South

Li Ping Chung

Australia

Myoung Ja Chung

Korea, South

Lucie Clapp

United Kingdom

Jocelyn Claude

United States of America

Gregory Conner

United States of America

Marco Contoli

Italy

Carlyne Cool

United States of America

Giuseppe M Corbo

Italy

Ferry Cornelissen

Netherlands

Jorge Correia-Pinto

Portugal

Peter Alan Coventry

United Kingdom

Bruno Crestani

France

Tore Curstedt

Sweden

Jeffrey Curtis

United States of America

Salvatore Cuzzocrea

Italy

Laura Dada

United States of America

Bhola Kumar Dahal

Germany

Sven-Erik Dahlén

Sweden

Ravi Dasu

United States of America

Ian Davis

United States of America

Francesco De Blasio

Italy

Stijn Piet De Langhe

United States of America

Roberto de Marco

Italy

Nicolas de Prost

France

Bart Dekkers

Netherlands

Marion Delcroix

Belgium

Phillip Dellinger

United States of America

Rolf Dembinski

Germany

Navneet Dhillon

United States of America

Y Peter Di

United States of America

Antonino Di Stefano

Italy

Alexander Dietrich

Germany

Quoc Thai Dinh

Germany

Claudio F Donner

Italy

James Donohue

United States of America

Wonder Drake

United States of America

Marjolein Drent

Netherlands

Paul Dudas

United States of America

Rodney Ehrlich

South Africa

Oliver Eickelberg

Germany

Hesham El-Shewy

United States of America

Jonas Eriksson

Sweden

David Erle

United States of America

Leo Fabbri

Italy

James Fabisiak

United States of America

Aurelie Fabre

Ireland

Joachim Fandrey

Germany

Neda Farahi

United Kingdom

Malgorzata Farnik

Poland

Heinz Fehrenbach

Germany

Charles Feldman

South Africa

Niki Fens

Netherlands

Lucas Fernandez

United States of America

Daniel Clark Files

United States of America

Bernie Fischer

United States of America

Anne Fitzpatrick

United States of America

Paul Forsythe

Canada

James Frank

United States of America

Charles Frevert

United States of America

Alex Fu

United States of America

Nobukazu Fujimoto

Japan

Jennie Gane

United Kingdom

Christine Garcia

United States of America

Francisco García-Río

Spain

Eric Gartman

United States of America

Gail Gauvreau

Canada

Moyar Qing Ge

United States of America

Thomas Geiser

Switzerland

Pierangelo Geppetti

Italy

Maher Ghamloush

United States of America

Saeid Ghavami

Canada

Yong Song Gho

Korea, South

Sudip Ghosh

United Kingdom

Peter Gerard Gibson

Australia

M Ian Gilmour

United States of America

Thomas Glaab

Germany

Constantinos Glynos

Belgium

Elena Goncharova

United States of America

Peter Gondrie

Netherlands

Reinoud Gosens

Netherlands

Kymberly Gowdy

United States of America

Andrew Greening

United Kingdom

Silvia Gregori

Italy

Timm Greulich

Germany

Cortland Griswold

Canada

Mathieu Gruet

France

Andreas Guenther

Germany

Tillie-Louise Hackett

Canada

Angela Haczku

United States of America

Stig Hagstad

Sweden

Andrew Halayko

Canada

Aaron Hamvas

United States of America

Meilan Han

United States of America

Keqi Han

China

Masayuki Hanaoka

Japan

Tomohiro Handa

Japan

Phil Hansbro

Australia

David Hansell

United Kingdom

Kevan Hartshorn

United States of America

Noboru Hattori

Japan

Gregory Hawkins

United States of America

Enrico Heffler

Italy

Dirk Henrich

Germany

Pieter Hiemstra

Netherlands

Yuji Higashimoto

Japan

Sandra Hodge

Australia

Cory Hogaboam

United States of America

Anne Holland

Australia

Olaf Holz

Germany

Robert Homer

United States of America

Guochang Hu

United States of America

Steven Huang

United States of America

Yu-Jing Huang

United States of America

Rudolf M. Huber

Germany

Alice Huertas

France

David Hui

Hong Kong

Marc Humbert

France

Machteld Hylkema

Netherlands

Robert Hyzy

United States of America

Masakazu Ichinose

Japan

Kaori Ihida-Stansbury

United States of America

Machiko Ikegami

United States of America

Dominique Israel-Biet

France

Kazuhiro Ito

United Kingdom

Shingo Iwano

Japan

Tae Jung Jang

Korea, South

Ilona Jaspers

United States of America

Marc Jeschke

Canada

Anton Jetten

United States of America

Samithamby Jeyaseelan

United States of America

Zhilong Jiang

United States of America

Zhi-Cheng JING

China

Martin Johnson

United Kingdom

Paul Jones

United Kingdom

Guy Joos

Belgium

Andreas Jung

Switzerland

Ravi Kalhan

United States of America

Yoshihide Kanaoka

United States of America

Min-Jong Kang

United States of America

Ferenc Karpati

Sweden

Atsushi Kato

United States of America

Ruth Katz

United States of America

Joseph Katz

United States of America

Steven Kawut

United States of America

Klaus Kenn

Germany

Eitan Kerem

Israel

Huib A M Kerstjens

Netherlands

Kwang Kim

United States of America

Yoon-Keun Kim

Korea, South

Dong Soon Kim

Korea, South

Paul Kingma

United States of America

Jason Kirkness

United States of America

Alex KleinJan

Netherlands

Joel Kline

United States of America

Darryl Knight

Canada

Alan Knox

United Kingdom

Beate Koch

Germany

Andreas Rembert Koczulla

Germany

Melanie Königshoff

Germany

Stephanie Korn

Germany

Gabor Kovacs

Austria

Krzysztof Kowal

Poland

Michael Kreuter

Germany

Markus Krupp

Germany

Olga Krysko

Belgium

Hilmar Kuehl

Germany

Ilko Kuljanac

Croatia

Wolfgang Kummer

Germany

Ken Kunisaki

United States of America

Han-Pin Kuo

Taiwan

Om Kurmi

United Kingdom

Emel Kurt

Turkey

Paige Lacy

Canada

Dominique Lagadic-Gossmann

France

Vincent Lagente

France

Timmy Lahm

United States of America

Vibha Lama

United States of America

Kevin Lamote

Belgium

Irene Lang

Austria

Peter Lange

Denmark

Sophie Lanone

France

Alexander Larcombe

Australia

François Laurent

France

William Lawson

United States of America

Aili Lazaar

United States of America

Romain Lazor

Switzerland

Ahmed Lazrak

United States of America

Maria Cristina Lebre

Netherlands

David Lederer

United States of America

Yong Chul Lee

Korea, South

Peter Lee

United Kingdom

Bruce Levy

United States of America

Christiane Lex

Germany

Gang Liu

United States of America

Shing-Hwa Liu

Taiwan

Marek Lommatzsch

Germany

Renaud Louis

Belgium

Kaizhi Lu

China

Tracy Luckhardt

United States of America

Amie Lund

United States of America

Lennart Lundblad

United States of America

Daoxin Ma

China

Harm Maarsingh

Netherlands

Rajiv Machado

United Kingdom

Roberto Machado

United States of America

Serdia Mack

United States of America

Tania Maes

Belgium

Helgo Magnussen

Germany

Toby Maher

United Kingdom

Sarah Maher

United Kingdom

Toby Maher

United Kingdom

Arnaud Mailleux

France

Mario Malerba

Italy

Patrick Mallia

United Kingdom

David Mannino

United States of America

Lin Mantell

United States of America

Francois Marchal

France

James Martin

Canada

Christian Martin

Germany

Sadis Matalon

United States of America

Thais Mauad

Brazil

Robin McAnulty

United Kingdom

Christine McCusker

Canada

Evert Meijer

Netherlands

Andrea Melani

Italy

Barbro Melgert

Netherlands

Keith C Meyer

United States of America

Tatiana Michel

Luxembourg

Marc Miravitlles

Spain

Firdaus A A Mohamed Hoesein

Netherlands

Lyn Moir

Australia

Paolo Montuschi

Italy

Jerome Moreaux

France

Peter Morfeld

Germany

Yasuo Mori

Japan

Danielle Morse

United States of America

Rory Morty

Germany

Joachim Mueller-Quernheim

Germany

Joaquim Mullol

Spain

Jeffrey Munson

United States of America

Jeffrey Myers

United States of America

Robert Naeije

Belgium

Yasutaka Nakano

Japan

Patrick Nana-Sinkam

United States of America

Viswanathan Natarajan

United States of America

Giuseppe Novelli

Italy

Benjamin Nwosu

United States of America

Anne O’Donnell

United States of America

Benjamin Odry

United States of America

Shinichiro Ohshimo

Japan

Horst Olschewski

Austria

Josuel Ora

Italy

Pierluigi Paggiaro

Italy

Mikell Paige

United States of America

Robert Paine

United States of America

Lena Palmberg

Sweden

Annie Pardo

Mexico

Anand Patel

United States of America

Ray Stokes Peebles

United States of America

Riccardo Pellegrino

Italy

Tonio Pera

United States of America

Jesus Perez-Gil

Spain

Michael Peters

United States of America

Irina Petrache

United States of America

Alejandro Pezzulo

United States of America

Victor Pinto-Plata

United States of America

Massimo Pistolesi

Italy

Carole Planes

France

Laurent Plantier

France

Don Poldermans

Netherlands

Venerino Poletti

Italy

Jill Poole

United States of America

Celeste Porsbjerg

Denmark

Nicolas Pottier

France

Y S Prakash

United States of America

Antje Prasse

Germany

Alice Prince

United States of America

Mirella Profita

Italy

Luis Puente-Maestu

Spain

Ville Pulkkinen

Finland

Lianqun Qiu

United States of America

Kurt Racke

Germany

Madeleine Rådinger

Sweden

Irfan Rahman

United States of America

Arjun Ram

India

Eve Ramery

Belgium

Sarah Rand

United Kingdom

Lee Reichman

United States of America

Niels Reinmuth

Germany

Luca Richeldi

Italy

Carlos Robalo Cordeiro

Portugal

Graham Roberts

United Kingdom

Mac Robinson

United States of America

Roberto Rodriguez-Roisin

Spain

Debra Romberger

United States of America

Eva Rönmark

Sweden

Mary Rose

United States of America

Robbert Rottier

Netherlands

Lewis Rubin

United States of America

Stephen Ruoss

United States of America

Kristina Rydell-Tormanen

Sweden

Bernhard Ryffel

France

Zeenat Safdar

United States of America

Matthias Salathe

United States of America

Daniel Salerno

United States of America

Dedmer Schaafsma

Canada

Andreas Schmid

United States of America

Lynn Schnapp

United States of America

Hildegard M. Schuller

United States of America

Malcolm Sears

Canada

Sanjay Sethi

United States of America

Satish Sharma

United States of America

Huahao Shen

China

Yoko Shibata

Japan

Larissa Shimoda

United States of America

Hiroaki Shimokawa

Japan

Vladimir Shirinsky

Russian Federation

Nikos Siafakas

Greece

Mujgan Sidal

Turkey

Philip Silkoff

United States of America

Jodie Simpson

Australia

Dave Singh

United Kingdom

Baljit Singh

Canada

Valérie Siroux

France

Brigita Sitkauskiene

Lithuania

Chrysanthi Skevaki

Greece

Trude Duelien Skorge

Norway

Hortense Slevogt

Germany

Peter Sly

Australia

David Smith

United Kingdom

Saul Soberanes

United States of America

Matteo Sofia

Italy

Paolo Spagnolo

Italy

Antonio Spanevello

Italy

Anita Spanjer

Netherlands

Mark Spears

United Kingdom

Lissa Spencer

Australia

Domenico Spina

United Kingdom

Michael Stalvey

United States of America

Bruce Stanton

United States of America

Charlie Strange

United States of America

Takafumi Suda

Japan

Hisatoshi Sugiura

Japan

Mary Sunday

United States of America

Erna Sziksz

Hungary

Ruth Tal-Singer

United States of America

Claudio Tantucci

Italy

Donald Tashkin

United States of America

Dries Testelmans

Belgium

Victor Thannickal

United States of America

Dirk Theegarten

Germany

Neil Thomson

United Kingdom

David Thornton

United Kingdom

Peter Tomassen

Belgium

Kjell Torén

Sweden

Dimitrios Toumpamakis

Greece

Gerard M Turino

United States of America

Silvia Ulrich

Switzerland

Soban Umar

United States of America

Paul Upton

United Kingdom

Isabelle Vachier

France

Dominique Valeyre

France

Thys van der Molen

Netherlands

Marco van der Toorn

Netherlands

Frederik-Jan Van Schooten

Netherlands

Jeroen Vanoirbeek

Belgium

Ruud Veldhuizen

Canada

Johanna Vendelin

Finland

Per Venge

Sweden

Giulia Veronesi

Italy

Giovanni Viegi

Italy

Gary Visner

United States of America

Claus Vogelmeier

Germany

Robert Voswinckel

Germany

Nicolas Vuilleumier

Switzerland

Elizabeth Wagner

United States of America

Benoit Wallaert

France

Patrick Walsh

Ireland

Garry Walsh

United Kingdom

Xiangdong Wang

China

Gang Wang

China

David Warburton

United States of America

George Washko

United States of America

Henrik Watz

Germany

Michael Wegmann

Germany

Athol Wells

United Kingdom

Fu-Qiang Wen

China

Christine Wennerås

Sweden

Thomas Wessendorf

Germany

Carl White

United States of America

Mark Wielpuetz

Germany

Martin Witzenrath

Germany

Will Wodzig

Netherlands

Keith Keat Huat Wong

Australia

Prescott Woodruff

United States of America

Weining Xiong

China

Ian Yang

Australia

Ivana Yang

United States of America

Mordechai Yigla

Israel

Cuneyt Yilmaz

United States of America

Hamid Zand

Iran

Andrea Zanini

Italy

Jacques Zimmer

Luxembourg

Jaroslaw Zmijewski

United States of America

Graeme Zosky

Australia

